# Towards verifiable cancer digital twins: tissue level modeling protocol for precision medicine

**DOI:** 10.3389/fphys.2024.1473125

**Published:** 2024-10-23

**Authors:** Sharvari Kemkar, Mengdi Tao, Alokendra Ghosh, Georgios Stamatakos, Norbert Graf, Kunal Poorey, Uma Balakrishnan, Nathaniel Trask, Ravi Radhakrishnan

**Affiliations:** ^1^ Department of Chemical and Biomolecular Engineering, University of Pennsylvania, Philadelphia, PA, United States; ^2^ Department of Bioengineering, University of Pennsylvania, Philadelphia, PA, United States; ^3^ In Silico Oncology and In Silico Medicine Group, Institute of Communication and Computer Systems, School of Electrical and Computer Engineering, National Technical University of Athens, Zografos, Greece; ^4^ Department of Pediatric Oncology and Hematology, Saarland University, Homburg, Germany; ^5^ Department of Systems Biology, Sandia National Laboratories, Livermore, CA, United States; ^6^ Department of Quant Modeling and SW Eng, Sandia National Laboratories, Livermore, CA, United States; ^7^ Department of Bioengineering, University of Pennsylvania, Philadelphia, PA, United States; ^8^ Department of Mechanical Engineering and Applied Mechanics, University of Pennsylvania, Philadelphia, PA, United States

**Keywords:** multiphysics models, machine learing algorithms, verification validation uncertainty quatification, model interpretability and forecasting, agent based models

## Abstract

Cancer exhibits substantial heterogeneity, manifesting as distinct morphological and molecular variations across tumors, which frequently undermines the efficacy of conventional oncological treatments. Developments in multiomics and sequencing technologies have paved the way for unraveling this heterogeneity. Nevertheless, the complexity of the data gathered from these methods cannot be fully interpreted through multimodal data analysis alone. Mathematical modeling plays a crucial role in delineating the underlying mechanisms to explain sources of heterogeneity using patient-specific data. Intra-tumoral diversity necessitates the development of precision oncology therapies utilizing multiphysics, multiscale mathematical models for cancer. This review discusses recent advancements in computational methodologies for precision oncology, highlighting the potential of cancer digital twins to enhance patient-specific decision-making in clinical settings. We review computational efforts in building patient-informed cellular and tissue-level models for cancer and *propose a computational framework that utilizes agent-based modeling as an effective conduit to integrate cancer systems models that encode signaling at the cellular scale with digital twin models that predict tissue-level response in a tumor microenvironment customized to patient information.* Furthermore, we discuss machine learning approaches to building surrogates for these complex mathematical models. These surrogates can potentially be used to conduct sensitivity analysis, verification, validation, and uncertainty quantification, which is especially important for tumor studies due to their dynamic nature.

## Introduction

Cancer is a heterogeneous disease with malignant cells that dynamically change their molecular signatures. The tumor tissue thus has a diverse collection of genetically distinct tumor sub-populations in a non-uniform spatial distribution. Each genotype exhibits different sensitivity to treatments. Spatial and temporal heterogeneity contribute to the development of resistance against therapies, complicating the effectiveness of treatments (Zhu et al., 2021; Vitale et al., 2021). In addition to genetic diversity, epigenetic modifications like DNA methylation, histone modifications, and chromatin remodeling can also drive therapeutic resistance (Vitale et al., 2021; Sadida et al., 2024). Combinatorial therapies targeting not only the drug/treatment sensitive tumor sub-clones but also the other drug-resistant subclones are likely to improve therapeutic responses (Zhu et al., 2021).

The tumor microenvironment also plays an important role in driving the evolution of cancer cells. It can lead to metabolic reprogramming in response to the availability of growth factors, oxygen, nutrients, drug exposure, and even mechanical cues such as cell density and extracellular matrix stiffness (Demicco et al., 2024). In the tumor microenvironment, stromal cells and immune cells exhibit substantial heterogeneity (Vitale et al., 2021), which further affects overall cancer heterogeneity.

Advances in single-cell sequencing (Baslan and Hicks, 2017; Lei et al., 2021) and multiomics (He et al., 2023; Menyhárt and Győrffy, 2021) techniques have enabled genetic, epigenetic, and even metabolic profiling of cancer cells at a cellular resolution, helping to delineate intratumoral heterogeneity (Lee et al., 2021; Schmidt et al., 2021; Ortega et al., 2017). Extensive multimodal data analysis on sequencing data has successfully identified biomarkers and stratifying risk in clinical settings (Lee et al., 2021; Schmidt et al., 2021; Ortega et al., 2017; Subramanian et al., 2020). Several multi-omic data repositories have been instrumental in furthering data-driven research to study cancer heterogeneity (Subramanian et al., 2020). (TCGA (NCI, 2022), TARGET (dbGaP, 2024), CPTAC (NCI, 2024), CCLE (CCLE, 2024), EUREKA (Peyser et al., 2022), AllofUs (Denny et al., 2019)) They are essential in guiding patient-specific treatment choices based on individual tumor profiles. However, these techniques on their own are not enough to decipher mechanisms or pathways that lead to the observed subclones. Moreover, these techniques are insufficient to characterize chemical or physical gradient-induced extrinsic spatial tissue heterogeneity. Physics-driven mechanistic systems biology modeling, which includes models of biochemical and genetic pathways using ordinary or partial differential equations, boolean networks, and even spatiotemporal models like hybrid cellular automaton and agent-based simulations, is essential. There is, however, a need to establish a verifiable computational framework that can utilize multi-modal patient-specific clinical information at different scales - molecular, single-cell sequencing, multi-omics, tissue histology, etc., in addition to physics-based and systems biology models to make tissue-level predictions. Such models can be used to explore model parameter space, simulate different treatments and compare model outcomes, formulate and test hypotheses to explore mechanisms of sub-clonal evolution, and elucidate pathways for tumor malignancy, sub-clonal evolution, resistance, and metastases.

A digital twin, as defined by the National Academies of Sciences, Engineering, and Medicine, is a set of virtual information constructs that mimics the structure, context, and behavior of a natural or engineered system, dynamically updated with data from its physical counterpart, with predictive capabilities to inform decision-making (National Academies, 2024). Cancer, as a complex and adaptive disease, requires personalized treatment strategies tailored to each patient’s genetic and environmental factors. Cancer cells’ ability to evolve and resist therapies further complicates treatment. To address this, an in-silico tumor simulator is needed, capable of capturing tumor complexity and updating with changes in the patient’s condition. Digital twin frameworks should incorporate mechanistic models at the cellular and tissue levels, continuously updated with real-time clinical and multiomics data. These cancer digital twins enable virtual patient simulations, allowing clinicians to test clinical hypotheses and refine therapeutic strategies in a predictive and personalized manner.

Multiscale hybrid digital twin models (virtual replicas of real-world entities) can facilitate personalized therapeutic strategies that are finely tuned to the unique characteristics of each patient. *Agent-based modeling can serve as an effective conduit for integrating cancer systems models that encode signaling at the cellular scale into digital twin models that predict tissue-level responses in a tumor microenvironment customized to patient cohorts.* Agent-based modeling (ABM) operates on a fundamental principle that each agent (e.g., a cell in a tumor) is programmed to follow a set of user-defined rules. These rules dictate how agents interact with each other and respond to their environment, thereby capturing the complex, dynamic behaviors observed in biological systems (Bonabeau, 2002). ABM frameworks are useful for cancer tissue systems because they can in corporate diversity at the single-cell level and predict the collective complex behavior of the tumor tissue spatiotemporally (West et al., 2023). These tissue-level spatiotemporal agent-based models can be powered by physics-based cellular systems biology models specific to different types of tumors, leveraging patient-specific clinical information. In this review, we examine various mechanistic cellular models and tissue-level agent-based models documented in the cancer research literature. We cite representative examples and explore potential strategies for integrating cellular-scale models with tissue-level frameworks.

**FIGURE 1:**
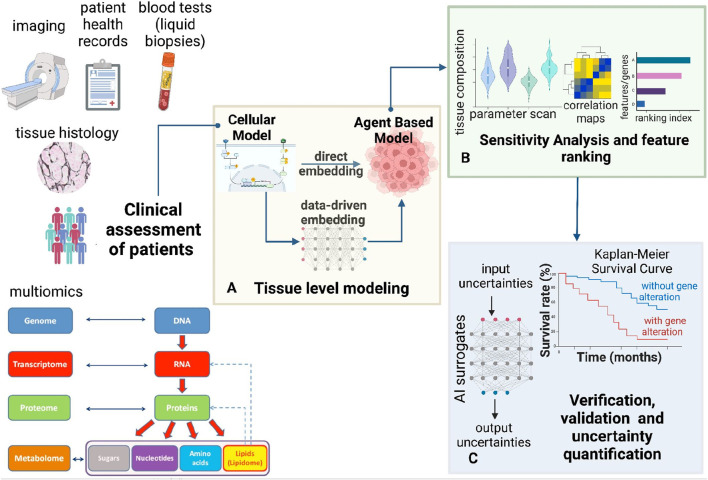
Modeling framework for multi-scale multi-agent spatiotemporal models. This figure presents a comprehensive bottom-up framework for hybrid multi-scale modeling. **(A)** This framework integrates cellular systems biology models with multi-agent simulations to predict tissue-level spatiotemporal dynamics. Cellular models, including ODE-based and Boolean approaches, can be embedded within the multi-agent framework to inform agent decisions. Cellular models can be constrained using patient-specific clinical information. Machine learning techniques can replace massive cellular models to enhance the efficiency of multi-agent simulations. Thus, the cellular models can also be embedded by training ML surrogates on data from the mechanistic cellular models. **(B)** Once the model is built, sensitivity analysis and feature importance assessment can be conducted **(C)** To ensure the robustness of the multi-scale framework, verification, validation, and uncertainty quantification (VVUQ) can be incorporated using AI surrogates. Clinical exploration using Kaplan-Meier survival analysis can help validate observations from the constructed hybrid model **(A)** and sensitivity analysis **(B)**.

Though such a hybrid modeling framework can be extremely useful for capturing the complexity of a tumor, the computational expense can grow exponentially as one increases the number of cells in the agent-based simulation. Machine learning approaches can reduce the computational expense of ABMs, conduct feature ranking, global sensitivity analysis, and uncertainty quantification (UQ) leveraging data-driven methods. Such a framework is outlined in Figure 1. While ML can help manage stochasticity and heterogeneity in ABMs and generate statistical measures for complex systems, the integration can result in limited interpretability due to the “black box” nature of ML models (Sivakumar et al., 2022). The synergy between ML and ABM leverages their respective strengths, but finding the right balance and integration strategy is complex and requires careful consideration of each approach’s limitations.

### Cellular systems biology models to encode cell behavior

Cancer cells undergo multi-faceted changes in the complex network of pathways, thus differentiating them from normal cells. These networks involve gene regulation, signaling, metabolism, and other dynamic pathways (Laubenbacher et al., 2009) responsible for cancer cells’ superior replicative potential, sufficiency in growth signals, insensitivity to growth signals, evading apoptosis, and even improved angiogenesis (Hanahan and Weinberg, 2000). Additional hallmarks that are generic to all cancer types include reprogramming of energy metabolism and evading immune destruction (Hanahan and Weinberg, 2011). Mathematical models have been built for these pathways in the past couple of decades, and rigorous analyses have been conducted to delineate mechanisms and simulate and test hypotheses. With the influx of multi-omics data, such models can be customized to patient information to improve predictability.

At the most fundamental level, cell behavior is governed by growth-inducing pathways and their counterpart apoptotic pathways. These two mechanisms work together to maintain normal cellular growth conditions. Alterations in these pathways are the most prominent indications of malignancy. A common pathway frequently studied in cancers is mitogenic signaling. The ErbB signaling pathways (ErbB receptor-mediated Ras-MAPK and PI3K-AKT pathways) have been modeled using mass action kinetics to study the effect of epidermal growth factor (EGF), heregulin (HRG) and other ligands in cancer cell mitogenesis (Chen et al., 2009; Ebata et al., 2022; Kirouac et al., 2013). A cell cycle progression and apoptotic pathway frequently implicated in several cancers is the p53-Mediated DNA damage response pathway. Multiple studies have conducted boolean network modeling and molecular state transition dynamics to understand oscillatory behavior, regulation and identify potential therapeutic strategies (Choi et al., 2012; Wang, 2013; Gao et al., 2020). In addition to these competing mitogenic and apoptotic pathways, certain additional pathways can become important to study in specific cancer types. For instance, the cross-talk between PI3K and androgen receptor (AR) signaling pathways promotes pathogenesis and treatment resistance in prostate cancer (Crumbaker et al., 2017). Moreover, as understanding of cancer mechanisms has advanced, phenotypic plasticity and epigenetic reprogramming have emerged as additional factors responsible for malignant transformation (Hanahan, 2022). Newer hallmarks highlight the need for models that incorporate these additional pathways, which are crucial for predicting cancer cell behavior.

It has become increasingly apparent that single pathway-specific models are insufficient for high-fidelity cell fate predictions. Combining these models and enabling dynamic interaction/cross-talk can be useful in predicting the cellular macrostates from the individual microstates of pathway-specific species (e.g., growth factors, tumor suppressors, etc.,) concentrations. Hybrid modeling protocols are gaining traction in building multi-pathway informed cancer cell state models. A recent publication from the CHIC Project Consortium (CHIC, 2017) describes a molecular model (as a plug-in component of a cancer digital twin) that combines the ErbB Receptor-Mediated Ras/Raf/MAPK and PI3K/AKT pathways (Chen et al., 2009) with p53-Mediated DNA damage-response module (Choi et al., 2012) by establishing interaction between the two models via the state of the common nodes, namely, Erk, Akt and PTEN (Kolokotroni et al., 2024).

Cellular models can predict phenotypes like cell growth and death probabilities, and cell growth in response to radiotherapy, chemotherapy, and hormonal therapy treatments. They can be tailored to patient-specific clinical data for personalized modeling. These models can help develop insights into individual cancer cell behavior but are not enough to gain insight into the collective behavior of tissues with heterogeneous cell types and cell-cell interactions. These models can, however, power tissue-level spatiotemporal models to build a holistic representation of a complex tumor microenvironment.

### Multi-agent models for simulating tissue-level spatiotemporal dynamics

The dynamics of cancer makes it important to capture and predict the spatiotemporal trends in tumor growth to develop effective treatment strategies. There has been an increasing interest in building agent-based models for cancer systems. Agent-based models consist of individual entities called agents in a shared environment. Agents can represent cells in a tissue or sections of blood vessels that reside in a space. These agents (cells) are programmed to act according to the rules set by the modeler (Cogno et al., 2024). Such rules encompass the cell-cell, cell-environment, and intra-cellular interactions. Cellular (i.e., agent) decisions can be based on physics-based models or probabilistic rules. When multi-cellular systems are allowed to evolve spatiotemporally based on these governing rules, the collective complex behavior of the system can be captured.

Agent-based models can be simulated using lattice-based or off-lattice methods. On-lattice (Lattice-based) models are spatially discrete models, where agent positions are asynchronously updated using simple migration rules. Such modeling style could lead to restrictions in simulating biologically close population dynamics. Lattice-based (On-lattice) methods include cellular automata models (where each lattice is occupied by one agent), lattice gas cellular automaton models (where each lattice site can contain multiple agents), and Cellular Potts models (CPMs) (use multiple lattice sites to represent each cell) (Metzcar et al., 2019). Off-lattice models usually simulate dynamics of individual agents using Newton equations of motion. These models provide more freedom in simulating behaviors like cell motility and chemotaxis. However, simulating these models is computationally expensive and the computational complexity increases with the number of agents. Off-lattice methods involve models that track the center of mass/volume of each agent and cell boundary-tracking methods (Metzcar et al., 2019). The latter is better suited for coupling detailed cell mechanics to fluid and solid tissue mechanics (Metzcar et al., 2019). Nava-Sedeno et al. (2020) and Metzcar et al. (2019) review these cell-based ABM methods with specific applications in cancer biology.

ABMs can simulate cancer initiation and progression by incorporating mechanistic cellular models informing each cell’s fate. They can also model tumor-host interactions, nutrient diffusion, hypoxia, angiogenesis, immune cell dynamics, and tumor-immune interaction (Cogno et al., 2024). It is extremely difficult to have mechanistic models to simulate all cellular behaviors. While constructing a multiscale hybrid model, one would need to approximate certain behaviors for which mechanistic models do not exist. ABMs thus become a powerful framework in complex multi-scale systems. ABMs can combine physics-based models (e.g., cell growth rate, death rate) with stochastic rule-based processes (where mechanistic molecular pathway-based models are currently non-existent). Examples of such phenotypes could be cell-cell adhesion, cell-matrix adhesion, chemotaxis, cellular uptake of drugs, hypoxia, angiogenesis, etc.

In the past two decades, numerous in-house and adapted ABMs have been developed to simulate tumor progression (Wang et al., 2015; Enderling et al., 2009; Norton et al., 2017; Yu and Bagheri, 2020; Rejniak, 2007) and its dependence on intra-tumor genetic diversity (Anderson et al., 2006), vasculature (Mehdizadeh et al., 2013; Yu and Bagheri, 2021; Cai et al., 2011; Duswald et al., 2024; de Montigny et al., 2021), nutrient (Kuznetsov and Kolobov, 2023) and oxygen (Anderson et al., 2006; de Montigny et al., 2021; Bull et al., 2020) (hypoxia and necrosis) gradients, solid stress (Kuznetsov and Kolobov, 2023), tumor-immune interactions (Heidary et al., 2020; van Genderen et al., 2024; Cess and Finley, 2020; Norton et al., 2019; Bergman et al., 2021), therapies like radiation therapy (Powathil et al., 2016; Chaudhuri et al., 2023), immunotherapy (Ruiz-Martinez et al., 2022; Gong et al., 2017) and even pre-clinical preventative vaccination studies (Palladini et al., 2010). Available open-source ABM software include PhysiCell (Ghaffarizadeh et al., 2018) (an off-lattice ABM framework that uses BioFVM (Ghaffarizadeh et al., 2016) to simulate diffusion partial differential equations for chemical substrates), BioDynaMo (Breitwieser et al., 2022), Chaste (Mirams et al., 2013; Cooper et al., 2020), CompuCell3D (Swat et al., 2012), and EPISIM (Sütterlin et al., 2013). These have been instrumental in improving the accessibility of multi-agent, multi-scale simulations for the computational biology modeling community.

### Embedding cellular models for decision-making in a multi-agent modeling framework

#### Differential equation based and boolean approaches for cellular modeling

Most systems biology models that simulate intracellular or molecular processes in a cell include continuous time, nonlinear and coupled deterministic or stochastic ordinary differential equations (ODEs). These can be solved using numerical discretization methods. COPASI (Hoops et al., 2006) is a commonly used open-source complex pathway simulator that supports systems biology markup language (SBML) representation of cellular biochemical networks. Many ABM packages (e.g., Chaste (Mirams et al., 2013), CompuCell3D (Swat et al., 2012), and EPISIM (Sütterlin et al., 2013)) support SBML to include systems of ODEs that simulate molecular pathways in individual cells (Metzcar et al., 2019). Thus, each cell/agent can have embedded cellular models informing cellular behavior (Rikard et al., 2019; Corti et al., 2021; Schuetz et al., 2013). In pathways where quantitative kinetic data of the constituent chemical species and reactions is unavailable or large-scale networks need to be modeled that are computationally expensive, a discrete boolean approach (Hemedan et al., 2022) is more feasible to extract qualitative insights. PhysiCell (Ghaffarizadeh et al., 2018) has an auxiliary boolean modeling package, MaBoSS (Stoll et al., 2017), that simulates intracellular pathways or molecular mechanistic models at a single-cell level.

#### Machine learning approaches to speed up multi-agent simulations

ABMs informed by cellular mechanistic models can be used to understand how intracellular changes can influence tissue-level behavior in individual cells. Complex intracellular models can have tens to hundreds of equations with hundreds to thousands of parameters. Thus, embedding mechanistic ODE-based or boolean models to inform the decisions of every agent at each time step in the dynamic simulation can be computationally very expensive. Surrogate models are data-driven models trained on data generated from high fidelity models that are computationally expensive to run. Surrogates can produce new simulation data faster and at a very little computational cost as compared to the original high-fidelity models. Such techniques might be extremely valuable in our hybrid modeling framework. The cellular mechanistic models can be used to generate data for training ML surrogates. Such surrogates can be used for predicting agent behavior instead of running the expensive mechanistic cellular models. An example of such use case in cancer agent-based modeling has been proposed by Cess and Finley (2020) proposed a novel data-driven technique to replace mechanistic models with pre-trained neural networks. They used an intracellular signaling model that informs macrophage phenotypes to produce simulation data. This generated data was used to train a simple input/output model using a neural network with cytokine concentrations in the microenvironment as input and macrophage differentiation state (M1 or M2) as output. In the ABM simulation, each macrophage agent samples the local cytokine concentrations (solved by diffusion PDEs coupled with the ABM) and decides its phenotypic fate based on the neural network. Such data-driven cellular embedding can significantly increase the computational speed. In case of absence of mechanistic models, machine learning models trained on clinical data can also be used to inform agent behavior (Sivakumar et al., 2022).

### Sensitivity analysis and feature importance in the multi-scale hybrid model

Predictive models are invaluable in biological research; however, they often lack sufficient experimental data to determine model parameters or initial conditions accurately (Zi, 2011). Building robust patient-specific predictive models requires longitudinal data from multi-omics, tissue imaging, and biopsies. However, in retrospective modeling efforts, access to such comprehensive data is often limited. Even when predictive modeling runs alongside clinical trials, challenges persist. These include uncertain data acquisition due to patient dropouts, failure to capture long-term post-trial data, and variability in data collection caused by non-standardized protocols across trial sites. These issues hinder the ability to create accurate and reliable models. In many instances, these values are estimated, thus leading to uncertainties that propagate through the model and affect its predictions. Sensitivity analysis techniques quantify the effect on fluctuations in the model output as caused by changes/uncertainties in the model inputs (Zi, 2011; Global Sensitivity Analysis, 2008). In the hybrid modeling context, these model inputs include model parameters, boundary conditions and initial conditions for cellular and tissue level models. Sensitivity analysis is used for evaluating robustness of mathematical surrogates of biological systems (Streif et al., 2016), parameter estimation (Linden et al., 2022), and rank the most important model parameter subsets that have the greatest impact on model outputs (Li et al., 2010). Such insights can also be used to design better experimental studies for the given biological system (Zi, 2011; Qian and Mahdi, 2020). Two types of sensitivity analyses exist - local and global (Table 1). Local sensitivity analysis (LSA) assesses the effect of small changes in individual model parameters on the output, varying one parameter at a time. On a wide range of values, parametric sensitivity analyses can be done on the multiscale hybrid agent-based model by scanning a few orders of magnitude for the parameter of interest. Global sensitivity analysis (GSA) evaluates the impact of varying all parameters simultaneously across their entire ranges, providing a comprehensive understanding of parameter influence and interactions. This method is more suited for highly nonlinear and coupled systems. Parameter sampling methods like Monte-carlo sampling and Latin hypercube sampling are required to conduct GSA. GSA includes variety of techniques - Multi-parametric sensitivity analysis (MPSA), Partial rank correlation coefficient (PRCC) analysis, Morris sensitivity analysis, Weighted average of local sensitivities (WALS), Sobol sensitivity analysis, Random sampling high-dimensional model representation (RS-HDMR), Fourier amplitude sensitivity test (FAST) (Zi, 2011). Zi (2011) discusses the advantages and disadvantages of all sensitivity analysis methods in the context of systems biology models. Global sensitivity methods are computationally expensive, even for simple models. They are extremely difficult to implement on hybrid models. Thus, there is scope for developing novel approaches to conducting global sensitivity analysis on complex hybrid multiscale models.

**TABLE 1:** Summary of sensitivity analysis types, including relevant methods, pros and cons of each analysis type, and a few examples of method use cases.

Type	Methods	Pros	Cons	A few examples of use cases
Local Sensitivity Analysis	Local derivative, One-way, Multi-way	Simple implementation, low evaluation cost	Only valid for small perturbations, May not capture interactions between parameters	Cancer models: Non-small cell lung cancer Kolokotroni et al. (2016), Advanced Gastric Adenocarcinoma Wen et al. (2016), Lung Cancer Screening Mahadevia et al. (2003), Solid Tumors Sefidgar et al. (2014) Other models: Aquatic Ecosystem Stressors Mondy et al. (2016), Electrical Power systems Qin et al. (2023)
Global Sensitivity Analysis	Sobol’ Indices, Morris Method, FAST/eFAST, PRCC, WALS, MPSA	A quantitative measure of sensitivity, Considers interactions, Provides a comprehensive view of parameter importance	Generally more computationally expensive, Requires a large number of model evaluations	Cancer models: Non-small cell lung cancer Kolokotroni et al. (2016), microbicide PK models Jarrett et al. (2016) Other models: Chemerin based anti-inflammatory treatment simulations Laranjeira et al. (2017), Liver radiofrequency ablation Hall et al. (2015), Electrical Power systems Qin et al. (2023)

#### Surrogate models for sensitivity analysis and feature importance

Surrogate models are data-driven approximations trained on outputs from high-fidelity models, which are often computationally expensive to execute. By providing rapid predictions with minimal computational effort, surrogate models offer a more efficient alternative to the original high-fidelity simulations. Traditionally, GSA requires sampling of points in the model input parameter space. The complex models need to be evaluated at these sampled points. The corresponding outputs are used to define a measure of sensitivity. Direct use of such sampling methods for GSA on complex models is often unaffordable due to high computational costs. Surrogates offer a practical alternative (Cheng et al., 2020). Surrogate models can approximate complex mechanistic models, thus reducing the computational costs associated with global sensitivity analysis (Cheng et al., 2020). They are developed using machine learning data-driven regression techniques (Tsokanas et al., 2022). The traditional surrogate models include polynomial regression and response surface models, Kriging/Gaussian process, polynomial chaos expansion, and Multivariate Adaptive Regression Splines (Cheng et al., 2020; Alizadeh et al., 2020). Supervised learning algorithms like neural networks (Liu et al., 2021; Li et al., 2016; Yang et al., 2021), random forests (Dasari et al., 2019), and support vector machines (Pruett and Hester, 2016) have also been used to build surrogate models and conduct sensitivity analysis.

One of the major goals of global sensitivity analysis techniques is selecting the most important model features that have the most impact on the model output. Traditionally, feature selection has long been used as a pre-processing step while building large multi-parametric data-driven models. The objectives of feature selection include building simpler and more comprehensible models, improving data-mining performance, and preparing clean, understandable data (Li et al., 2017). Li et al. (2017) classify feature selection methods into these categories: similarity-based, information-theoretical-based, sparse-learning-based, and statistical-based. In addition to data-driven models, these methods can also be exploited to extract important features from complex mechanistic models like ODE systems, boolean networks or even hybrid tissue-level agent-based models. Identifying such features can provide meaningful insights into the important pathways for malignancy represented in the model. Since all feature selection algorithms require sampling through large input parameter spaces, computations can get very expensive with the complex multiscale hybrid models. Surrogates of these complex models can be used to perform feature selection. The variance-based Sobol global sensitivity method is among the most popular feature ranking methods. This variance-based technique determines the contribution of each model (or surrogate model) feature and their interactions with the overall variance of the target variable. It has been implemented for purely data-driven models as well (Zouhri et al., 2022; Fel et al., 2021; Efimov et al., 2017).

A game theoretic approach to feature ranking is Shapley value analysis. Shapley value (Winter, 2002) is a cooperative game theory approach used to distribute the total gain (or cost) among players based on their individual contributions. For feature attribution in machine learning models, each feature is considered a player in a coalition, and the Shapley value assigns a value to each feature representing its contribution to the model’s prediction. Features with higher Shapley values can be considered more important, aiding in selecting the most relevant features (Chen et al., 2023). Though this method is a straightforward check for global sensitivity, there are a few downsides: assumption of feature independence, difficulties with scalability, and high computational expense (Aas et al., 2021).

#### Clinical exploration of feature importance predictions

Sensitivity analysis methods can predict the top model parameter subsets that are responsible for aggressive tumor proliferation and metastatic tendencies, thus leading to poor patient prognosis. Once the most important model parameter subsets are identified, a simple check for clinical significance can be done by conducting Kaplan-Meier survival analysis on available clinical data. Worse survival in patient cohorts with alteration in the identified most sensitive model species would corroborate the model ranking results from the multiscale model.

### Verification, validation, and uncertainty quantification (VVUQ) of the multi-scale hybrid-modeling framework

Experimental data for validation are available in cancer data repositories like TCGA (NCI, 2022), TARGET (dbGaP, 2024), EUREKA (Peyser et al., 2022), and AllofUs (Denny et al., 2019). These sources have clinical information like mutations, RNA sequencing, or other forms of liquid biopsies such as ctDNA, miRNA, or exosome profiling. Relevant data from these repositories can be used to calibrate different sub-modules (cellular models, tissue level diffusion, cell-population ABM) in the hybrid model. The key predictions from the multi-scale hybrid model can be compared against or regressed with the measured biomarkers. The time evolution of key biomarker concentrations in the hybrid level model can be compared against experimental and clinical data where possible (eg., comparing predicted PSA levels with available patient PSA level data for prostate cancer studies) Spatial tissue-level ABM results can be compared with tumor imaging results, particularly, tumor spheroid diameters and aspect ratio measurements. Spatial tissue-level ABM results can be compared with tumor imaging results, particularly, tumor spheroid diameters and aspect ratio measurements. A caveat here is that most spatiotemporal modeling studies might not find enough relevant clinical information including imaging, multi-omics and biopsies, to sufficiently validate the models.

Hybrid multi-scale models can have high fidelity but can be computationally expensive owing to several sub-modules capturing the complexities of the system. Heterogeneous multiscale approaches can be used for cost-effective linking of the submodules via information transfer at specific time intervals (Ee et al., 2007; Kevrekidis et al., 2004). However, increasing model complexity complicates model validations and inferential understanding (Cogno et al., 2024). Additionally, there is a scarcity of patient-specific clinical data for training or validation of such complex models. To ensure these models are viable in a clinical setting, extensive validation of their predictions is necessary, along with quantification of the uncertainty in the predicted outputs based on the model inputs. Verified and validated GenAI-based digital twins can increase the size of the data cohort and improve the robustness of uncertainty analysis, providing reliable predictions (Kamruzzaman et al., 2024).

Mathematical models inherently provide approximations of real-world phenomena, necessitating verification processes to quantify numerical errors and ensure model accuracy. Uncertainty Quantification (UQ) goes a step further by assessing the range of possible values that a quantity of interest may take within a given problem. UQ accounts for inherent variability and uncertainties in model inputs, parameters, and structure, offering a more comprehensive understanding of the model’s predictive capabilities and the reliability of its outcomes. Roy and Oberkampf (2011) classify uncertainty in model predictions arising from three broad categories: (1) model inputs, (2) numerical approximations, and (3) assumptions in the mathematical model. Input uncertainties encompass variability in data and parameters. (1) Model input uncertainties encompass inherent variability in data and parameters. They can be classified as aleatory or epistemic. Aleatory uncertainties refer to the variability in model input due to inherent random effects. In cellular or tissue level systems biology models, aleatory uncertainties can include experimentally derived kinetic parameters for ODEs, and physiologically relevant initial conditions to simulate diffusion of nutrients, oxygen in the tissues. Epistemic uncertainty stems from incomplete knowledge about the system, particularly, input data or model parameters. Such uncertainties are usually explored through alternative assumptions. In systems biology models, unknown mechanisms can be treated as epistemic uncertainties, and the range of assumptions used to bridge each epistemic uncertainty can be evaluated. (2) Numerical approximation errors assess simulation accuracy associated with computational solving techniques (verification). (3) Assumptions in the mathematical model are usually made to keep the model tractable. These assumptions contribute to model form uncertainty.

Roy and Oberkampf (2011) present a practical approach for quantifying uncertainty using probability boxes (P-boxes). To generate the P-boxes, both aleatory and epistemic uncertainties are propagated through the model to generate an ensemble of cumulative distribution functions of model output quantity of interest. For model validation, model predictions are compared with experimental data to estimate model form uncertainties. These are treated as epistemic uncertainties. The final step in this approach involves determining the total uncertainty in the system response by combining P-boxes derived from input uncertainties, extrapolated model form uncertainties, and uncertainties due to numerical approximations (Roy and Oberkampf, 2011). This comprehensive approach delineates confidence in model predictions, ensuring robust and reliable assessments of the system’s performance. Even though this method is valuable for UQ in complex models, it can get computationally expensive with increase in number of uncertain model input parameters and model form complexity. Hybrid multiscale models in cancer are built using several approximations and simplifications of tumor heterogeneity, tumor–microenvironment interactions. All these could contribute to epistemic uncertainty. Moreover, calibration of certain cellular or tissue models to clinical and experimental data involves significant aleatoric uncertainty. This would lead to a huge computational burden. ML surrogates offer a promising avenue for enhancing VVUQ by efficiently approximating complex models and providing rapid sampling. There is considerable scope for improvement with further research into the mathematics of VVUQ and the use of ML surrogates to enhance VVUQ for complex models.

There is a critical need for benchmarks to ensure the safe and reliable use of computational models in real-world settings. These benchmarks would provide standardized criteria for model verification, validation, and performance, ensuring models meet the necessary requirements before deployment. The ASME V&V 40 standard (ASME, 2018), recognized by the FDA, offers a risk-based framework to establish the credibility of computational models used in medical device development. ASME also has VVUQ standards for various modeling techniques, including Computational Fluid Dynamics, Computational Solid Mechanics, and machine learning models. There are verification and validation benchmarks for digital twins for manufacturing applications as well (Bitencourt et al., 2024). However, a similar benchmark for physiological models developed for clinical applications has not yet been established. In clinical contexts, the development of a robust Verification, Validation, and Uncertainty Quantification (VVUQ) benchmark is crucial to ensure that these models are trustworthy and safe for use, as patient safety becomes the paramount concern.

## Discussion

### Building verifiable cancer digital twins for precision medicine

Cancer is characterized by its dynamic and evolving nature. As the disease progresses, the diversity within cancer cell populations typically increases (Jacquemin et al., 2022). Such diversity can lead to uneven distributions of genetically varied tumor cells across different areas of the tissue (known as spatial heterogeneity). It can also cause changes over time in the genetic composition of these cells (temporal heterogeneity). This heterogeneity is a key factor in developing treatment resistance, making precise evaluation of tumor heterogeneity crucial for effective treatments (Dagogo-Jack and Shaw, 2018). Technologies like multi-region sequencing, single-cell sequencing, autopsy sample analysis, and longitudinal liquid biopsy are advancing rapidly and hold significant promise for unraveling the intricate clonal structure of tumors (Lee et al., 2021; Ortega et al., 2017; Gilson et al., 2022). Nevertheless, the complexity of the data gathered from these methods cannot be fully interpreted through multimodal data analysis alone. Data-driven techniques can help uncover important pathways and give insights into important biomarkers of malignancy (Sathyanarayanan et al., 2020; Vaske et al., 2010). This crucial information can be leveraged to build relevant mathematical models incorporating the identified pathways. Mathematical models can incorporate physical laws and dynamic behaviors, allowing for more detailed insights into the mechanisms of cancer growth, treatment response and resistance. The predictive capability of mathematical models are superior to purely data-driven models as the former allow hypothesis testing via insilico clinical trials, leading to more informed decision making. Mathematical modeling is critical in integrating personalized data, providing the groundwork for creating verifiable cancer digital twin (VCDT) models that could revolutionize our approach to understanding and treating cancer (National Academies, 2024; Strobl et al., 2023). Physics based models can be informed using clinical or experimental data. In context of our proposed hybrid modeling framework, data can be integrated in different the sub-modules. For example, tissue imaging information can help initialized cell-population agent-based models (Cess and Finley, 2023); genomics, proteomics and other omics data can be used to identify, model and calibrate cellular pathways (Kolokotroni et al., 2024; Byrne et al., 2016). Physics-based modeling has effectively integrated data across different scales and physics to reveal mechanisms underlying functional emergence. In addition to the genetic spatial and temporal heterogeneity, physics-based spatiotemporal models are well-suited to represent extrinsic heterogeneity in the tissue microenvironment due to substrate gradients, signal gradients, etc., (Bray et al., 2019)

Integrating physics-based hybrid models with multi-modal data can increase the computational complexity of the system, thus increasing computational costs. The recent emergence of machine learning as a powerful method to integrate diverse data types and uncover correlations among complex phenomena offers significant potential. However, relying solely on machine learning can overlook fundamental physical laws, leading to poorly defined problems or unrealistic solutions. Machine learning can be used to enhance physics-based hybrid models by facilitating better data integration. Generative AI methods can produce synthetic data for physics-based model calibration and validation, in cases where real-world data is sparse (Kamruzzaman et al., 2024). ML methods can also be used to create simplified surrogates of physics-based hybrid models (Peng et al., 2021). Consequently, a multidisciplinary approach to creating a digital twin by integrating machine learning with physics-based modeling can provide new insights into disease mechanisms, identifying new targets and treatment strategies (Alber et al., 2019).

This review discusses physics driven methods to encode the mechanisms driving an evolving primary tumor personalized to the genotype of a real-world patient. We underline the importance of mechanistic, multiscale hybrid cellular models that can integrate core biological pathways universally connected to cancer hallmarks of continued proliferation and evasion of cell cycle checkpoints common to several tumors. In addition to these core modules, several tumor-specific and patient-specific pathways can also be added to drive context-specific cell decision-making in the tumor microenvironment. Such cellular physics-based models can be embedded in a tissue-level framework. ABMs are modular and can embed many physics-based, stochastic, or data-driven models to enhance biological accuracy. ABMs can be spatially initialized using high-resolution imaging and tissue histology information (Cess and Finley, 2023). They can leverage patient-specific omics data to calibrate models for patient-representative prediction. ABMs can thus provide insights into cancer biology that can guide experimental designs and patient-specific therapeutic strategies (Cogno et al., 2024).

In addition to the bottom-up, cancer biology-driven digital twin development, a top-down, clinical medicine-driven approach has emerged in the literature (Kolokotroni et al., 2024; Kolokotroni et al., 2016; Stamatakos, 2010; Ouzounoglou et al., 2017; Stamatakos et al., 2014). This approach aims to create digital twins designed to answer specific clinical questions, such as the optimal sequencing of chemotherapy and surgery for individual patients. A careful hybridization of both approaches can be highly effective for certain cases. Some recent examples of top-down digital twin models include Oncosimulator developed by Stamatakos (2014). It is an information technology system that simulates tumor responses to therapies. This system includes modules for multiscale data handling, image processing, and spatiotemporal simulations. It combines clinical data with advanced modeling techniques to predict tumor evolution and optimize personalized treatment strategies. Using similar principles, Oncosimulator (Ouzounoglou et al., 2017) for acute lymphoblastic leukemia was created using hybridization of discrete entity-discrete event-based (Stamatakos, 2010) spatiotemporal modeling with machine learning. This model leverages extensive patient transcriptomics data to enhance predictive accuracy.

Sensitivity analysis is a fundamental tool in studying biological systems. It quantifies the influence of parameter changes on system responses, aiding in identifying key model inputs driving output variations. In precision medicine, this understanding is instrumental in assessing disease prognosis and designing more effective therapies by elucidating critical factors influencing disease progression and treatment response. Global sensitivity analysis methods are conducted on mechanistic models to identify the most important model parameters and conduct robustness analysis (Whitacre, 2012) to determine stiff and sloppy modes in the system. In addition to the traditional methods, data-driven approaches are becoming increasingly popular for GSA and feature importance.

**FIGURE 2:**
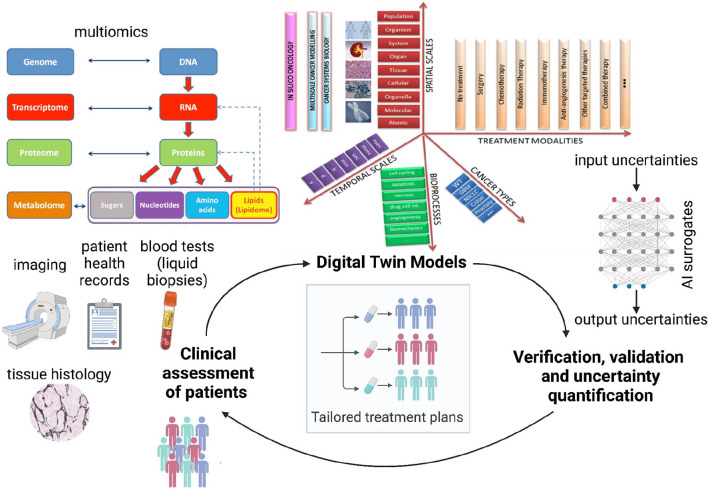
Proposed schematic of the Verifiable Cancer Digital Twin (VCDT) framework. The VCDT will enable dynamic, bidirectional information exchange between the patient and the digital twin, continuously updating with new clinical data. Digital twin models [illustration adapted from *CHIC consortium* (CHIC, 2017) *final report*] can span across temporal and spatial scales, include relevant bioprocesses, incorporate different treatment modalities, and simulate several cancer types. This adaptive physics-based model will integrate physical and data-driven approaches to represent the evolving tumor, generating synthetic data to enhance AI surrogate model training. Continuous Verification, Validation, and Uncertainty Quantification (VVUQ) will ensure robustness and reliability, supporting informed clinical decisions for personalized treatments.

The Digital Twin of a tumor of a real-world cancer patient aims to encode the dynamic, bidirectional interaction of the patient and a digital twin to inform clinical decisions regarding interventions, treatments, and assessments, which in turn updates the digital twin (National Academies, 2020). Data is gathered from the patient and the tumor through various clinical assessments to inform the digital twin, creating a virtual representation comprised of models that describe the temporal and spatial characteristics of the patient and tumor, with dynamic updates from real-world data (National Academies, 2020). Integrating interpretability through physics, biomarker alignment, cohort stratification, and cell state dynamics represents an aspirational approach to advancing precision medicine via digital twins. As the patient and tumor constantly evolve and the data collection can change over time, VVUQ must occur continually for digital twins (National Academies, 2020), see Figure 2. The VVUQ framework for the digital twin model consists of identifying and quantifying aleatory and epistemic uncertainties in the model inputs, verifying numerical approximation errors associated with the solvers, and quantifying uncertainties originating from the chosen form of the model by comparing model outputs with experimental data. It is essential to address UQ comprehensively, covering all aspects of the digital twin, including patient data, modeling and simulation, and decision-making processes (National Academies, 2020). There is a compelling need to develop novel methods to perform VVUQ in multiscale, dynamic digital twins. By addressing UQ comprehensively across patient data, modeling and simulation, and decision-making processes (National Academies, 2020), we can enhance the reliability and clinical viability of digital twin models.

### Data-driven methods for multi-modal model interpretability and forecasting

**FIGURE 3:**
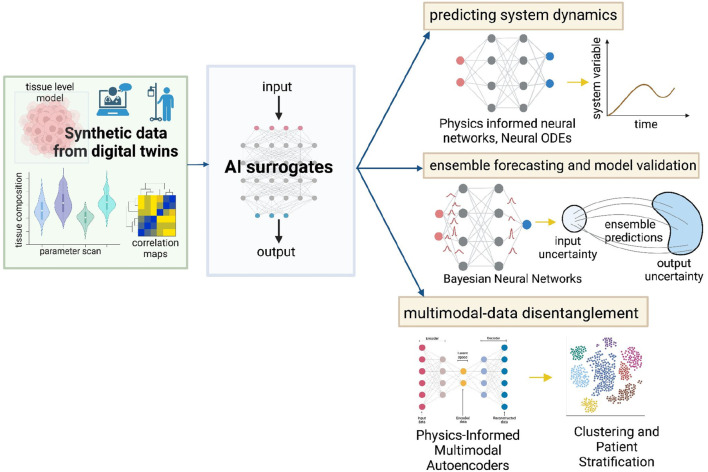
Data-driven methods to enhance utility and reliability of digital twins. Synthetic data generated from the digital twins can be used to construct AI surrogate models that capture data-informed dynamics while preserving the system physics - PINNs, Neural ODEs. Bayesian Neural Networks can enable ensemble forecasting and aid model validation. Additionally, Physics-Informed Multimodal Autoencoders can be used to disentangle multi-modal data and stratify patients. These techniques can improve personalized treatment strategies.

Digital twins are invaluable in simulating virtual tumor dynamics and treatment. In the process, these models can vigorously generate high-dimensional data, owing to the tissue-level ABMs and embedded cellular models. For every patient, the digital twin can provide temporal tumor evolution information, spatial tumor-immune or tumor-drug interactions, temporal concentrations of important biomarkers and much more. Sensitivity analysis and large parametric studies on digital twins can result in a treasure trove of synthetic training data for building supervised machine learning surrogates. This data can be used to drive several machine learning methods for improving VVUQ, risk-stratifying patient cohorts and predicting important cancer system dynamics (illustrated in Figure 3). The recent advancements in multi-modal (Baltrusaitis et al., 2019; Stahlschmidt et al., 2022; Ektefaie et al., 2023) and physics-informed (Cuomo et al., 2022; Jabbari Zideh et al., 2023) machine learning have significantly enhanced the ability to analyze complex, multimodal scientific datasets summarized in Figure 3. Physics-informed neural networks (PINNs) operate by embedding physical laws, expressed as partial differential equations (PDEs), into the training process of neural networks. This integration allows PINNs to leverage both observational data and underlying physical principles to infer solutions to complex problems (Cuomo et al., 2022; Karniadakis et al., 2021). In systems biology (Yazdani et al., 2020), PINNs are particularly useful for modeling biological processes by providing robust predictions even with limited and noisy data, facilitating the understanding of high-dimensional biological systems, and improving the accuracy of simulations. The extensive spatiotemporal data generated through parametric, aleatoric, and epistemic variations in the digital twin can provide an opportunity to implement innovative approaches to *capture data-informed dynamics* effectively. Computationally efficient data-driven models can be built to simulate tumor dynamics by utilizing sparse identification of nonlinear dynamics (Brunton et al., 2016) integrated with neural ordinary differential equations. Neural ordinary differential equations (Chen et al., 2019) (ODEs) can learn black-box dynamics and structure-preserving formalisms to capture key dynamics of course-grained variables like surface area, circularity, aspect ratio, and clustering index. For our suggested digital twin architecture, Neural ODES could conform to the physics of reaction, diffusion, and convection powering the core ABM and preserve the fluctuation-dissipation relationship when coupling the agent dynamics with the dynamics of fields.

Developing AI-enabled computationally efficient surrogate models will reduce computational overload while also aiding the implementation of robust methodologies for VVUQ. McCulloch et al. (2022) propose a novel framework for calibrating agent-based models (ABMs) by integrating History Matching (HM) and Approximate Bayesian Computation (ABC) (Toni et al., 2009). By first employing HM, the parameter space is reduced by ruling out implausible models, thus decreasing the computational burden. This is followed by using ABC to generate a detailed posterior distribution of parameters, incorporating quantified uncertainties. Bayesian Neural Networks (BNNs) (Neal, 1996) integrate Bayesian inference into neural networks to quantify uncertainties in model predictions. By treating the weights and biases of the neural network as probability distributions rather than fixed values, BNNs can account for both aleatoric uncertainty (inherent noise in the data) and epistemic uncertainty (uncertainty due to limited data). Such models can be used for reliable *ensemble forecasting* (Olivier et al., 2021). Additionally, multiple trajectory simulations can be done by direct sampling of multiple neural network parameters. The simulated data statistics can be validated by comparison with real data summary statistics and quantifying the residuals.

Multimodal synthetic data generated from the digital twin and available real-world clinical data can be disentangled and mapped to clinical stratification of patients based on risk, genetic footprint, and other drivers of population heterogeneity. One notable framework, the Physics-Informed Multimodal Autoencoders (PIMA) (Walker et al., 2024), presents a solution for *disentangling multimodal data*. PIMA fuses data into a multimodal posterior by embedding individual modalities into a shared latent space and utilizing a product-of-experts formulation. Integrating a Gaussian mixture model (GMM) prior further identifies shared features across different data modalities, while a mixture-of-experts decoder incorporates prior scientific knowledge (in the form of digital twin synthetic data), ensuring structured disentanglement of the latent space. This approach enables cross-modal generative modeling and inference and facilitates the discovery of high-dimensional features, overcoming traditional limitations related to high-fidelity measurement and characterization (Walker et al., 2024).

These advancements can significantly enhance the interpretability, forecasting capability, and mechanism identification of cancer digital twin models. AI-enabled verifiable cancer digital twins will facilitate the development of personalized treatment strategies across various cancer types, ultimately leading to more effective and tailored healthcare solutions. This integration of cutting-edge technologies holds the promise of revolutionizing cancer treatment and patient outcomes.

From a clinical perspective, emphasizing comprehensive and individualized data collection from single patients is crucial to ensure accurate computational modeling and avoid artificial or amalgamated data. Educating clinicians on necessary data during the patient journey, and ensuring collaboration among hospital information systems and external providers, is vital. Integrating diverse data sources such as social media, wearables, ePROs (electronic Patient-Reported Outcomes), and ePROMs (electronic Patient-Reported Outcome Measures) enhances data quality. Ensuring data privacy and security through federated learning, and addressing trust through clinical validation and randomized trials comparing digital twin model predictions with standard treatments, can foster acceptance and utilization in clinical decision support.

### Current limitations and future perspectives

Digital twins in oncology hold significant promise but face several challenges that need to be addressed before they can be widely adopted in clinical settings. In the past few years, there have been National Cancer Institute and US Department of Energy initiated collaborative exploratory projects to promote development of patient-specific cancer digital twins (Stahlberg et al., 2022). Hadjicharalambous et al. (2021) review recent cancer mathematical models in literature, with the potential for utility for insilico-clinical trials. Including image-driven models, radio/chemo-therapeutic planning models and tumor vasculature models. These models have demonstrated the potential of digital twins in oncology, while also highlighting key challenges that need to be addressed. One major limitation is the sparse and incomplete multimodal data currently available, which hinders the effective integration of patient-specific information into models. Advancements in AI could potentially help resolve this problem. Furthermore, continuously updating digital twins with real-time patient data remains a challenge. This requires the development of novel dynamic calibration methods to maintain accuracy and adaptability of digital twins in clinical applications.

Validation of digital twin model predictions is crucial and will need to be done through randomized clinical trials that compare these predictions with standard treatments. Additionally, for reliably exploring hypotheses and conducting *in-vivo* clinical trials, advancements in gaps in applicability of VVUQ methods to digital twins need to be addressed. As highlighted in this review, recent machine learning advancements, and others still emerging, will play a critical role in refining digital twin models and utilizing digital twin derived synthetic data to answer important clinical questions.

For digital twins to support clinical decision-making effectively, collaborations between cancer modelers, bioinformaticians, machine learning experts, mathematicians, and clinicians are essential. Clinicians must be educated on the necessary data required during a patient’s treatment journey, ensuring seamless data collection and integration. Hospital information systems, along with external medical service providers, need to work together to compile comprehensive and individualized data. In addition, the integration of diverse data sources, such as social media, wearables, ePROs (electronic Patient-Reported Outcomes), and ePROMs (electronic Patient-Reported Outcome Measures), will help enhance data quality and better support cancer modeling. However, concerns around data privacy and ownership must be addressed, requiring stringent benchmarks and regulatory frameworks to ensure patient data protection.

Similar to the ASME V&V40 guidelines for model verification and validation in medical devices, there is a need for standardized frameworks to ensure the safe and effective deployment of cancer digital twin models in clinical settings. Such guidelines will help establish clear protocols for verifying the accuracy, reliability, and clinical relevance of digital twins, ensuring their safe integration into patient care. Regulatory bodies like the FDA (US) and EMA (Europe) will need to develop clear guidelines for using digital twins in clinical trials, with the regulatory landscape likely to evolve continuously (Li, 2024). The scientific community must remain adaptable to these changes. Despite all these challenges, cancer digital twin technology has the potential to transform cancer medicine by accelerating drug development and reducing clinical costs. It could potentially become a cornerstone of future cancer treatment.

### Concise Summary

Cancer’s heterogeneity often undermines the efficacy of conventional treatments. Advances in multiomics and sequencing have provided insights, but the complexity of the data requires robust mathematical models for full interpretation. This review highlights recent advancements in computational methodologies for precision oncology, emphasizing the potential of cancer digital twins to enhance patient-specific decision-making. We propose a framework that integrates agent-based modeling with cellular systems biology models, utilizing patient-specific data to predict tissue-level responses. Additionally, we discuss machine learning approaches to build surrogates for these models, facilitating sensitivity analysis, verification, validation, and uncertainty quantification. These advancements are crucial for improving the accuracy and reliability of clinical predictions.
